# Parametric study on the geometrical parameters of a lab-on-a-chip platform with tilted planar electrodes for continuous dielectrophoretic manipulation of microparticles

**DOI:** 10.1038/s41598-020-68699-4

**Published:** 2020-07-16

**Authors:** Arash Dalili, Erfan Taatizadeh, Hamed Tahmooressi, Nishat Tasnim, Pamela Inés Rellstab-Sánchez, Matthew Shaunessy, Homayoun Najjaran, Mina Hoorfar

**Affiliations:** 1 School of Engineering, Faculty of Applied Science, The University of British Columbia, Kelowna, BC V1V 1V7 Canada; 2 School of Biomedical Engineering, Faculty of Applied Science, Faculty of Medicine, The University of British Columbia, Vancouver, BC V6T 1Z3 Canada

**Keywords:** Biomedical engineering, Lab-on-a-chip

## Abstract

Advances in lab-on-a-chip (LOC) devices have led to significant improvements in the on-chip manipulation, separation, sorting, and isolation of particles and cells. Among various LOC-based approaches such as inertia-based methods, acoustophoresis, and magnetophoresis, the planar-slanted-electrode dielectrophoresis (DEP) method has demonstrated great potential as a label-free, cost-effective, and user-friendly approach. However, the devices built based on this method suffer from low flow throughput compared to devices functioning based on other LOC-based manipulation approaches. In order to overcome this obstacle, the geometrical parameters of these types of DEP-based devices must be studied to increase the effectiveness of DEP manipulation. With the consideration of both numerical and experimental studies, this paper studies the geometrical factors of a LOC platform consisting of tilted planar electrodes with the goal of achieving higher throughput in continuous manipulation of polystyrene particles. COMSOL Multiphysics software was used to study the effect of the electrodes geometry on the induced electric field. The simulation results show that by increasing the electrode’s width and decreasing the electrode’s spacing, higher DEP force is generated. Furthermore, the experimental outcomes indicated that lower channel height, higher voltage, and larger particle size resulted in the most improvement to DEP manipulation. Additionally, the experimental results demonstrated that slanted electrodes with an angle of 8° with respect to the direction of flow provide a more effective configuration.

## Introduction

Cell manipulation, as a preliminary step for cell-based analysis, is a rapidly growing area of interdisciplinary research for the development of single-cell technologies. Over the past two decades, single-cell manipulation and analysis methods have improved significantly due to advances in microfluidic cell manipulation methods^1,2^. These methods can be broadly categorized as either passive or active. The passive methods, including microfiltration^3^, inertia-based^4^, contraction–expansion channels^5^, deterministic lateral displacement^6^, and pinched flow fractionation^7^, do not rely on any external forces. On the other hand, the active methods, such as dielectrophoresis (DEP)^8^, magnetophoresis^9^, acoustophoresis^10^, and optical-based manipulation^11^, require an external force to manipulate the cells/particles. Although passive methods can handle higher flow rates, active methods offer more control over cells/particles, real-time tuning, reliability, and higher manipulation efficiency^12–14^.

Generally speaking, DEP is a label-free method (with only a handful of studies using biomarkers to change the dielectric properties of cells^15^) which makes it more cost-effective compared to other active methods such as magnetophoresis, fluorescent activated cell sorters (FACS) and magnetic activated cell sorters (MACS). DEP-based devices are also easier to fabricate compared to other label-free active methods such as acoustophoresis. Besides, DEP has shown promising high-throughput capabilities with reported throughputs of 5,000 cells per second^16^, 10,000 cells per second^17^, and more recently 170,000 cells per second^18^. These advantages have attracted researchers towards the use of DEP for a range of applications such as cell patterning^19^, trapping^20^, focusing^21^, enrichment^22^, separation^23^, and isolation^24^.

The first DEP devices were designed to separate cells and particles in a batch process^14^. During this process, the sample fills a chamber that has electrodes on its surface that trap particles or cells affected by positive DEP (pDEP) within the chamber. As a result, by flowing the sample into the chamber, only the pDEP entrapped particles remain in the chamber, thereby separated from the rest of the particles. Later, by deactivating the electric field, the channel can be washed to collect the formerly trapped particles. Although this is a simple and effective technique, interest in pDEP has declined over the years due to its low throughput.

Continuous DEP-based lateral displacement of particles is another technique that has garnered attention due to its higher throughput ^25,26^. For this purpose, electrodes can be patterned into different 2D or 3D configurations (such as trapezoidal electrode arrays^27^, spiral electrodes^28^, liquid-based electrodes^29^, slanted interdigitated^30–32^ and interdigitated electrodes^33–35^), as well as insulator-based DEP^36^.

Slanted electrodes in 3D or planar forms have been used for manipulation purposes such as focusing and sorting since 1998^37^. Some researchers have taken advantage of pDEP to sort particles of different sizes, passing over slanted electrodes^38,39^. Despite these studies, the combination of nDEP and slanted electrode has been more commonly used. For instance, this method has been used for particle focusing^40^, particle sorting^41–44^, separation of breast cancer cells from blood cells^45^, enrichment of circulating nucleated cells from peripheral blood^46^, separation of mouse P19 embryonic carcinoma and red blood cells^47^ among others^48^. Overall, 3D electrodes can produce more uniform and stronger DEP forces along the channel. Yet, the difficulty of perfect alignment and sealing has hindered their mass production^31^. Planar electrodes, on the other hand, are more favorable as they can be fabricated by following a standard lithography process, which makes them simple, cost effect, and easy to operate in compared to 3D electrodes. Furthermore, the induced DEP force in such a 2D configuration could be strong enough to manipulate the particles or cells if the proper electrodes and microchannel sizes were employed.

To take advantage of the planar electrode design in the DEP manipulation, the geometry parameters and operational conditions of the chip (including voltage, channel height, electrode’s width, spacing, and angle) have to be optimized based on the size of the particles and the throughput of the device, accordingly. Several simulation and numerical models have been developed to investigate the effect of the planar electrode’s width and spacing^34,49–52^. The effect of electrode’s geometry on levitation of the particles^34^, the magnitude of the electric field above the electrodes^49^, and the variation of the electric field at the center and the edge of the electrode along the channel height^51^ have been reported. Although not specifically for the planar-shape electrodes, a few studies have reported the effect of the channel’s height on the levitation distance of particles^51^ and the particle capture time^34^. However, these effects depend on specific conditions and methods which are implemented in the previously reported studies, such as the geometry of the electrodes and the mode of the DEP force (nDEP versus pDEP). Kralj et al.^31^ developed a model to study the effect of several parameters, including the channel’s length, particle size, voltage, and flow rate, on the transverse displacement of the particles. They validated their model using experimental data; however, the effect of the channel height and electrode’s angle for DEP-based manipulation of microparticles using tilted interdigitated electrodes is not investigated yet.

This paper studies a continuous nDEP-based particle separator, which uses planar slanted electrodes. First, we conducted a numerical simulation to solve/model the electric field and calculate the DEP force caused by the electrodes. Then, the electrodes’ width and spacing were optimized to achieve the highest efficiency with the applied DEP force. Based on the simulation’s results, we fabricated the chip using soft lithography. Following fabrication, we conducted multiple experimental setups to investigate the effect of the particle size, applied voltage, channel’s height, and electrode’s angle on the effectiveness of the DEP-based particle manipulation. The results showed that the DEP force is more dominant for larger particles, and higher voltages (as expected). Additionally, it was shown that decreasing the channel’s height increases the hydrodynamic forces against the DEP force; yet the increase in the induced DEP force is more significant. Based on our results, it was shown that the 25-μm channel height and tilt angle of 8° provides the highest manipulation of particles in the slanted electrodes configuration.

## Design of the proposed device

**Figure 1:**
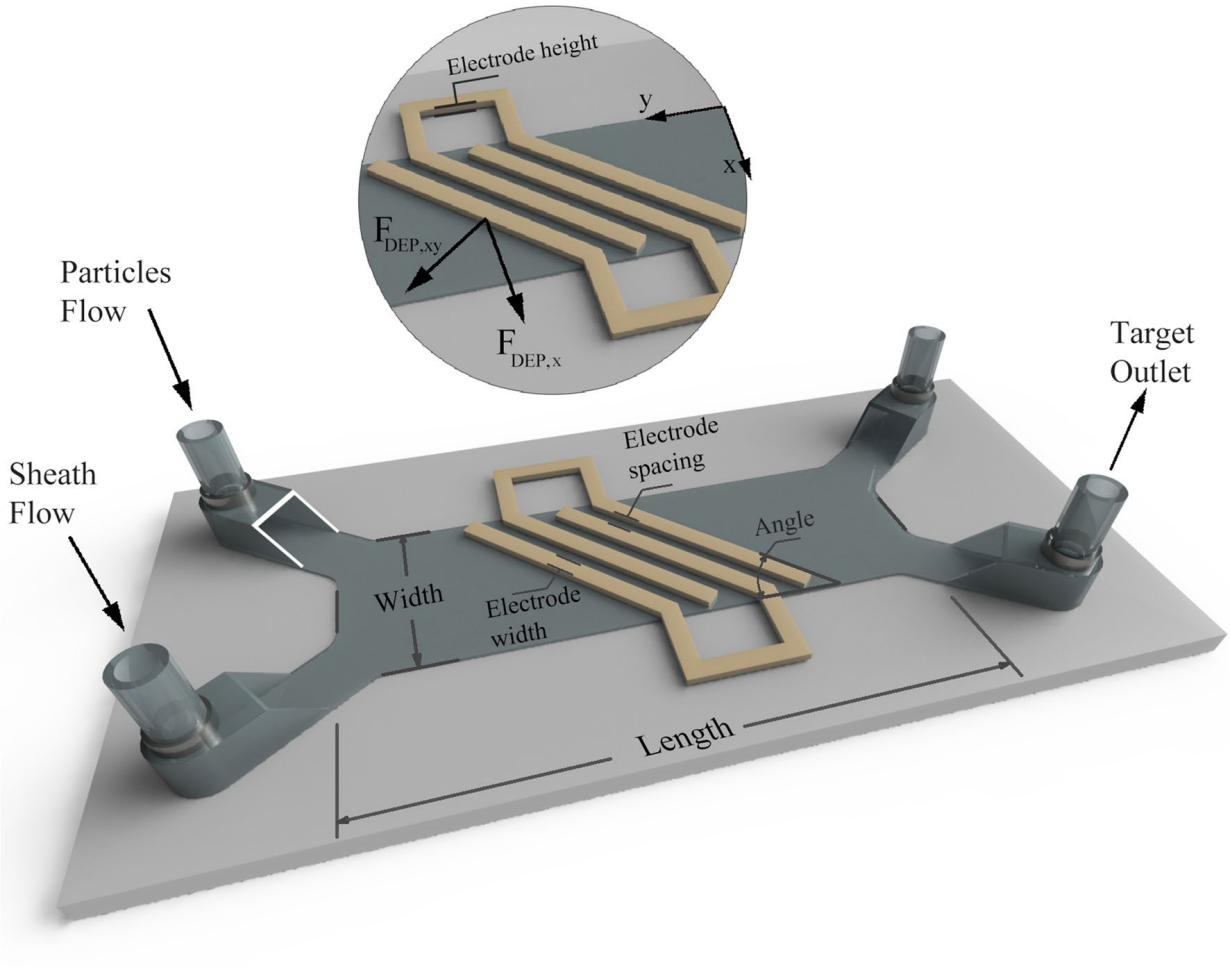
A schematic of the proposed DEP-based micro-separator with all its elements.

As it is shown in Fig. 1, the proposed DEP device consists of two inlets and two outlets. The particles enter the channel from one of the inlets and then they are pushed to the sidewall due to the hydrodynamic force caused by a sheath flow. As the particles approach the electrodes’ region, depending on their electrical properties and the resulting CM factor, they experience different amplitude and direction of DEP force. The DEP force, as shown in Fig. 1, consists of two components in the $XY$ plane. The lateral component pushes the particles toward the opposite side of the channel. If the DEP force is dominant against the hydrodynamic forces, the particles are deflected to the target outlet. Otherwise, they remain in the same lateral position and follow the laminar streamline and move to the other outlet. As a result, the particles can be separated based on their response to the DEP force. The parameters affecting this process include the size of the particles ($R$), electrodes’ width ($w$) and spacing ($g$), the height of the channel ($h$), voltage ($V$) and frequency ($f$) of the applied electric field, and the flow rate ($\dot{V}$).

## Results

### Simulation of the electric field

In order to apply an effective DEP force for cell/particle separation, the geometry of the electrodes must be optimized. The optimized design leads to the higher throughput and lower voltage (which means lower joule heating occurrence and higher cell viability). The geometrical parameters of this design, including the width of the electrodes and the electrode’s spacing, can affect $\nabla E^{2}$ (in Eq. (2)) and the resulting DEP force.

The input geometry for the simulation consists of two same-sized rectangular electrodes positioned at the bottom of the microfluidic channel. For several channel heights (25 µm, 40 µm, 60 µm, and 75 µm) the electric field was simulated for electrode’s spacing and width, ranging from 5 µm to 50 µm and 10 µm to 110 µm, respectively. An AC electric field of 2 V peak to peak was applied to the electrodes while the other boundaries of the channel were assumed to be isolated.

**Figure 2:**
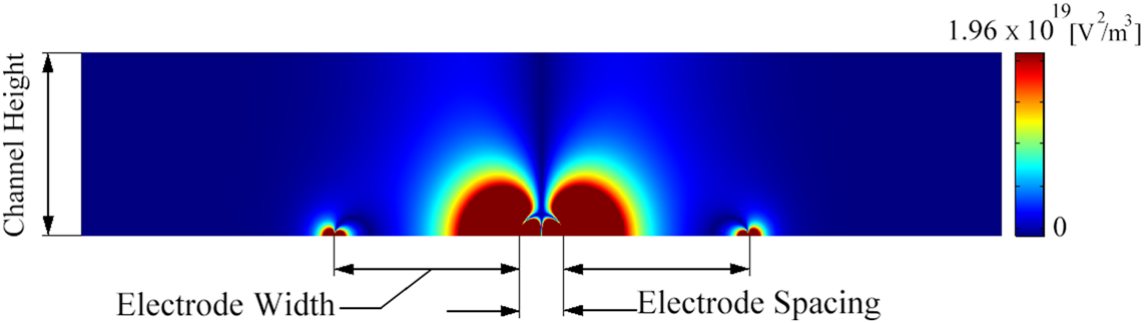
The geometry of the channel (used for the COMSOL Multiphysics simulation) and $\nabla E^{2}_{x}$ contour (the width of the electrodes is 100 µm and the electrode’s spacing is 15 µm).

In this study only the horizontal component of the gradient of the electric field square ($\nabla E^{2}_{x}$) is considered, as it is the dominant factor in the lateral displacement of the particles due to DEP force. Figure 2 presents the ${ }\nabla E^{2}_{x}$ counter for a sample with 100 µm electrode’s width and 15 µm electrode spacing. It shows that the highest gradient of the electric field is around the edges of the electrode. Increasing the distance from the electrodes leads to a decrease in the gradient of the electric field. As a result, if the channel is long enough, nDEP pushes the particles to the top of the channel, away from the electrodes, where the gradient of the electric field is minimum. As we study the nDEP effect in this research we have focused on the value of $\nabla E^{2}_{x}$ at the top of the channel. In deeper channels, for example, 75 μm, the cells do not necessarily reach the very top of the channel. However, since the optimum electrode width and spacing are not too sensitive to the height of the channel, we can neglect the actual height position of the cells and consider them within the top region of the channel. This misplacement, however, becomes greater as the height of the channel increases and should be considered as a factor in deeper channels.

**Figure 3:**
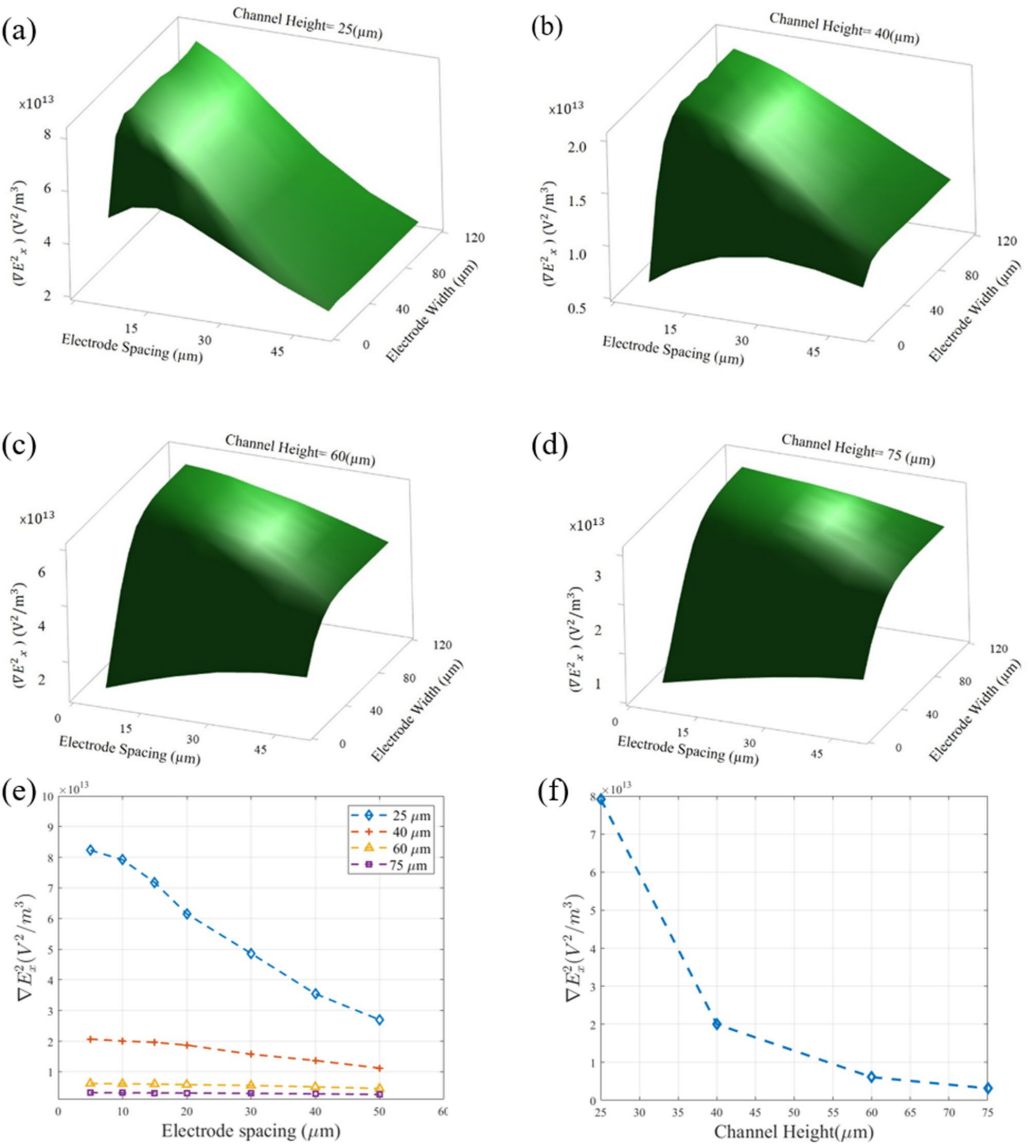
The combined effect of the electrode spacing and electrode width on $\nabla E^{2}_{x}$ for different channel heights: (**a**) 25 μm, (**b**) 40 μm, (**c**) 60 μm, (**d**) 75 μm (drawn with Minitab 19.2.0^54^). (**e**) $\nabla E^{2}_{x}$ for each one of the studied channel heights based on their chosen electrode width (drawn with MATLAB R2019b^55^). (**f**) The effect of the channel height on $\nabla E^{2}_{x}$ (drawn with MATLAB R2019b^55^).

The maximum values for $\nabla E^{2}_{x}$ at the top of the channel are calculated and shown in Fig. 3a–d. These results show that by increasing the width from 10 to 110 µm for any electrodes’ spacing and channel height values, the $\nabla E^{2}_{x}$ increases until it reaches a maximum level. From that point on, any further increase in the width does not change the value of $\nabla E^{2}_{x}$. This critical width is highly dependent on the height of the channel while only slightly dependent on the electrode’s spacing. Hence, for each channel height, designing the electrodes smaller than this corresponding critical width must be avoided. Based on these results, 40 µm, 40 µm, 60 µm, and 80 µm were chosen as the electrode width for 25 µm, 40 µm, 60 µm, and 75 µm channel heights, respectively. As the next step in analyzing the simulation results, the effect of the electrode’s spacing was investigated. Considering the aforementioned electrode widths for each channel height, the $\nabla E^{2}_{x}$ values were calculated for different channel’s heights and electrode’s spacing which is demonstrated in Fig. 3e. The results show that decreasing the electrode spacing leads to the higher values of $\nabla E^{2}_{x}$ for all channel’s heights. This effect is more significant for lower channel heights. For instance, decreasing the electrode spacing from 50 to 5 µm for the 25 µm and 75 µm channel’s heights led to almost 205% and 22% enhancement of the $\nabla E^{2}_{x}$, respectively. These results also reveal that decreasing the electrode spacing from 10 to 5 µm increases $\nabla E^{2}_{x}$ by less than 4%. Although greater values of $\nabla E^{2}_{x}$ are more favorable, considering the fabrication limitations, 10 µm was chosen as the electrode spacing to allow for easier fabrication, lower costs, and higher reproducibility.

After finding the design criteria for the electrodes, the optimum condition for each channel height was chosen based on the data presented in Fig. 3a–e. Using the chosen electrodes’ geometries, the effect of the channel’s height on $\nabla E^{2}_{x}$ was investigated and presented in Fig. 3f. It can be observed that the value of $\nabla E^{2}_{x}$ greatly increases by lowering the channel’s height. For instance, decreasing the height from 75 to 60 µm, 60 to 40 µm and 40 to 25 µm increased the $\nabla E^{2}_{x}$ by approximately 93%, 229%, and 295%, respectively. However, to have a full understanding of the effect of channel height on DEP-based manipulation of particles, the effect of the hydrodynamic forces must also be considered, which is presented in the next section.

### Experiments

Based on the simulation results, the geometry of the electrodes inducing the strongest DEP force was determined. However, the simulation only considered the DEP force and not the hydrodynamic forces caused by the flow of the particles in the device. In order to investigate the effect of the channel’s height, electrode angle, particle size, and applied voltage on the effectiveness of the continuous DEP manipulation, a series of experiments were conducted, which are presented in this section.

As demonstrated in the previous section, the DEP force decreases as a particle moves away from the electrodes, meaning that lowering the channel height leads to a greater DEP force. Another force on the particles that varies with the channel height is the drag force. This force, which is caused by the velocity difference of the particles and their surrounding fluid can be found by using Eq. (1):

1 $$F{ }_{drag} = { }6{ }\pi { }\mu { }R{ }v$$

where $\mu$ is the viscosity of the medium, $v$ is the fluid velocity relative to the particles and $R$ is the radius of the particles. When the DEP force dominates over the drag force, the particles are moved laterally towards the target outlet, shown in Fig. 1. As the height of the channel decreases, the particles are expected to be more influenced by the DEP force. On the other hand, decreasing the channel height reduces the cross-sectional area of the channel. Hence, in order to keep the volumetric throughput of the system constant, the velocity should increase, which leads to a larger drag force on the particles. Also, higher velocities lead to larger particle Reynolds number, which means that the inertial lift and lateral forces become more effective. Other forces such as the interaction of the particles with each other as well as the channel’s surfaces, can also affect the displacement of the particles. For instance, Brownian motion plays a role in the trajectory of the particles; however, it is not a dominant force for the sizes of the particles that are studied in this research.

In order to investigate the effect of the channel’s height on the combination of all the aforementioned forces, and as a result the DEP-based manipulation of the particles, several experiments were conducted. To eliminate the effect of the size of the particles on the CM factor, the angular frequency was set to 6 MHz (at this frequency, the CM factor was almost -0.477 for all the studied [(see Supplementary Fig. S1 online)]). While the length (10 mm) and width (700 μm) of the channel, and the deflection angle of the electrodes (5°) were kept constant, the experiments were run for different channel heights (25 μm, 40 μm, 60 μm, and 75 μm). For each height, the goal was to find the maximum flow rate (volumetric throughput) as a function of the applied voltage that results in the particle collection efficiency of more than 99%. In other words, for each voltage, the flow rate was increased up to the point at which any further increase in the flow rate resulted in missing the particles in the desired outlet.

**Figure 4:**
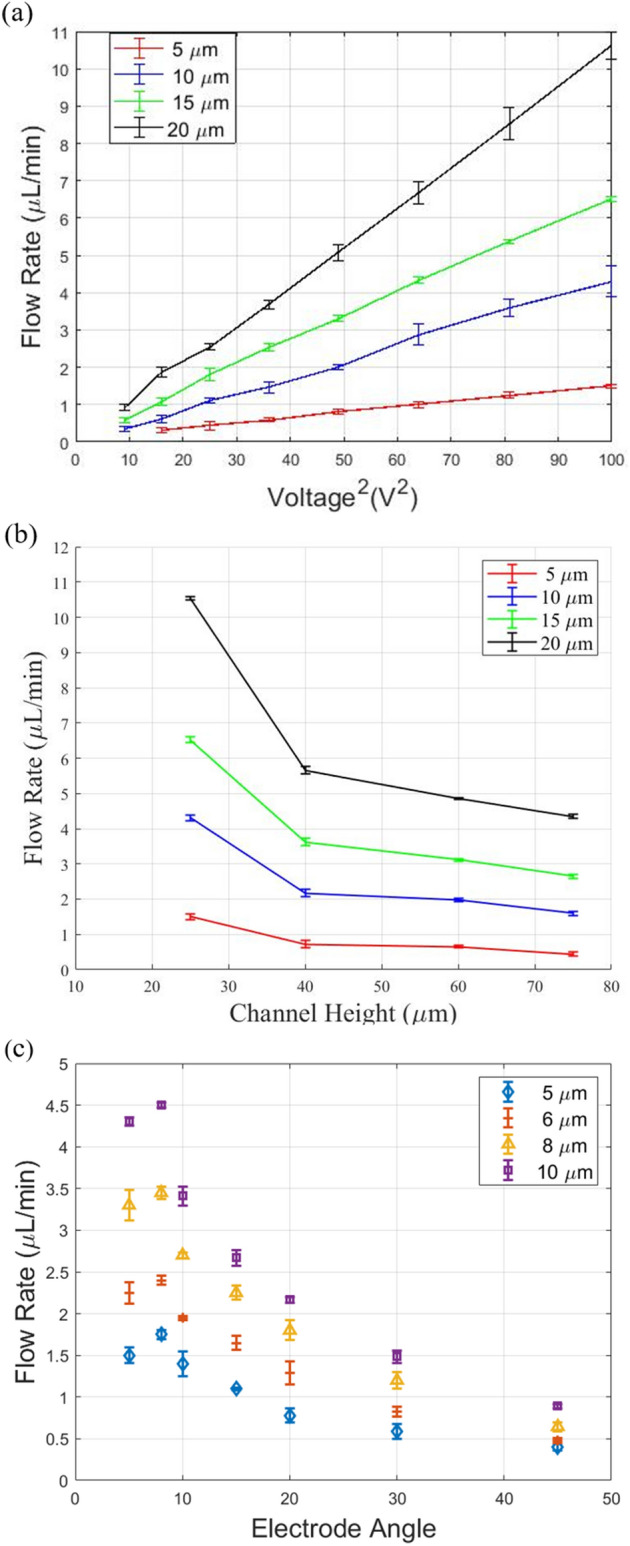
(**a**) The effect of the applied voltage squared on the volumetric throughput for a 25-μm channel height. (**b**) The effect of the channel height on the volumetric throughput for an applied voltage of 10 Vpp. (**c**) The effect of the electrode angle on the flow rate (drawn with MATLAB R2019b^55^).

Four different diameter sizes of the particles (5 μm, 10 μm, 15 μm, and 20 μm particles) and a voltage range of 2–10 V (peak to peak) were tested. In order to make sure that the experiments were repeatable, we repeated each of the tests three times. According to Eq. (2), the DEP force is proportional to the gradient filed squared, resulting in a linear relation with the DEP force and voltage squared. Consequently, the effect of the voltage squared is a representative of all the affective parameters in the DEP force, specifically the permittivity of the medium and the CM factor. As the size of the particles contributes to both the DEP and hydrodynamic forces, its effect is separately investigated. The average volumetric throughputs have been presented with their standard deviation (shown as the error bars) in Fig. 4a to show the effect of the voltage squared on the maximum effective volumetric throughput for four different particle sizes and a 25 μm micro-channel height. As expected, the larger the particles, the easier it is to deflect them with DEP force, and hence, the higher the flow rate. The same trend was observed for deeper channels. Figure 4b compares the maximum flow rates obtained from the largest voltage for different particle sizes as a function of the channel height. The results posit that as the height increases from 25 to 75 μm, the volumetric throughput decreases (for all particle sizes) since the DEP force becomes less dominant.

Another important parameter to consider is the angle of the electrodes. As mentioned earlier, the lateral component of the DEP force moves the particles laterally. This component is represented in Fig. 1. In essence, the lower the angle, the larger the lateral component of the DEP force. On the other hand, for a fixed length of the channel, increasing the electrode’s angle increases the number of the electrodes that can fit in the channel, which may cause greater overall DEP-based manipulation of the particles.

To investigate this, we conducted experiments on the effect of the electrode angle on the DEP manipulation of polystyrene particles with various diameters. Previously, we showed the effect of the particle size on the throughput, with an increment of 5 μm in size. In order to fine-tune the increment and study the effect on the throughput, we switched to 5, 6, 8, and 10 μm particles for this part. A range of electrode angles from 5° to 45° were fabricated and tested. The 5° design comprised only one pair of electrodes; while the 45° consisted of 24 pairs. The frequency and voltage were fixed at 6 MHz and 10 V, respectively. Similar to the previous results obtained for the effect of the channel height, the highest flow rate affecting the particles effectively was determined for each one of the electrode angles, and particle size. Each experiment was repeated three times to make sure that the results are reproducible. The average values are presented in Fig. 4c and the standard deviation of the results are depicted by using the error bars. The results showed that the size of the particles has a significant effect on the response. Besides, decreasing the angle from 45° to 8° increases the strength of DEP-based manipulation. However, any further decrease to the angle below 8° weakened the DEP manipulation of the particles.

## Discussion

In this paper, we investigated the effect of the electrodes’ geometry and channel height on DEP-based manipulation of particles. Based on the simulation results, we showed that increasing the electrode’s width to a certain amount, called critical width, causes greater DEP force. Further increase of the width above the critical value did not change the maximum DEP force. We also demonstrated that the critical width is dependent on the height of the channel. Furthermore, electrode spacing also affects the DEP force. Decreasing the electrode spacing, strengthen the induced DEP force. The effect of decreasing the electrode spacing on the induced DEP force is more significant for lower channels. Based on the simulation results, the induced DEP force can increase up to 198% by proper selection of electrode width (110 μm) and spacing (10 μm) for a 75 μm channel height.

Experimental results showed that the DEP manipulation is stronger for larger particles at higher voltages. A linear relationship was exhibited between the voltage squared and the volumetric throughput of the system. Since the strength of the DEP force is linearly related to the *CM* factor and voltage squared, the same linear relation between the *CM* factor and the volumetric throughput is expected. Decreasing the absolute value of the *CM* factor, e.g. through manipulation of the frequency of the AC voltage, is expected to have the same effect as decreasing the voltage squared, which will lead to lower volumetric throughput.

Additionally, the tests revealed that the channel height inversely affects the effectiveness of the DEP-based manipulation; meaning that lowering the height can lead to higher volumetric throughput of the system. For instance, decreasing the channel height from 75 to 25 μm, increases the throughput by up to 241% for 5 μm particles. The last parameter that we optimized was the electrode angle. The trend of the effective throughputs of the system showed that generally lower angles are more effective, and the optimum angle is around 8°. Taken together, this work provides a guideline for designing the optimum electrode width and electrode spacing to achieve the highest induced DEP force for the proposed electrode configuration. In addition, the size-based response of the proposed device to changes of the electrodes deflection angle as well as the channel height was experimentally tested and discussed. These findings can help to achieve the optimal geometrical and operational conditions for designing effective DEP-based devices.

## Methods

### Theory of DEP

The DEP force can be generated when a non-uniform electric field polarizes particles and medium. The DEP force on a particle depends on the electrical properties of that particle relative to the medium. The time-averaged DEP force on a spherical cell or particles can be calculated by the Eq. (2):

2 $$F_{DEP} = 2\pi \varepsilon_{m} R^{3} Re\left( {CM} \right)\left( {\nabla E^{2} } \right)$$

where $\varepsilon_{m}$ is the electrical permittivity of the medium, *R* is the radius of the particles, *E* is the electric field, and *Re(CM)* is the real part of the Clausius–Mossotti factor. The magnitude and sign of DEP force depend on the electrical properties of that particle relative to the medium which is expressed as CM factor:

3 $$CM = \frac{{\varepsilon_{p}^{*} - \varepsilon_{m}^{*} }}{{\varepsilon_{p}^{*} + 2\varepsilon_{m}^{*} }}$$

where $\varepsilon^{*}$ is the complex permittivity, $p,$ and $m$ subscripts are notations for the particles and medium, respectively. The complex term of permittivity can be found by:

4 $$\varepsilon^{*} = \varepsilon - i\frac{\sigma }{\omega }$$

where $\sigma$ is the conductivity, $\omega$ is the angular frequency of the applied electric field, and *i* is the imaginary component. The absolute permittivity of a substance can be calculated by:

5 $$\varepsilon = \varepsilon_{0} \varepsilon_{r}$$

where $\varepsilon_{0}$ is the permittivity of the free space ($\varepsilon_{0}$ = 8.854 × 10^–12^ F/m), and $\varepsilon_{r}$ is the relative permittivity of the substance.

Based on the sign of the *CM* factor, DEP force can be categorized into two groups of positive dielectrophoresis (pDEP) and negative dielectrophoresis (nDEP). In pDEP, the particles are more polarizable than their surrounding media. In this case, the direction of the DEP force is towards the region with the highest intensity of the electric field gradient. On the other hand, the higher polarizability of the medium in comparison to the particle causes the nDEP effect where pushes the particles towards the region with the lowest electric field gradient.

In this study, we have investigated the effect of DEP on polystyrene particles. The conductivity of the polystyrene particles can be found by the following equation:

6 $$\sigma_{e} = 2\frac{{K_{s} }}{R} + \sigma_{b}$$

where $K_{s}$ is the surface conductance (which is 10^–9^ S for polystyrene particles), $\sigma_{b}$ is the bulk and $\sigma_{e}$ is the effective conductivity. The conductivity of the media and bulk conductivity of the polystyrene particles are 2.03 × 10^–3^ S/m and 10^–15^ S/m, respectively. The *CM* factor for different sizes of the polystyrene particles was calculated (see Supplementary Fig. S1 online).

### Simulation of the electric field

The numerical simulation was developed using a commercialized finite element analysis (FEA) software package (COMSOL Multiphysics, version 5.3a). Since the Z direction (along the microchannel’s length) of DEP force was negligible compared to X and Y directions (along the width and height of the microchannel), only a cross-sectional view of the microchannel was simulated. Hence, the 2D computational domain was chosen which consisted of two rectangular electrodes, which were positioned at the bottom of the microchannel.

The electrical field inside the fluid domain was evaluated based on the Gauss’s law, Ohm’s law, and continuity equation for the current. The governing equations in a frequency domain form are expressed as:

7 $$E = - \nabla V$$

8 $$D = \varepsilon_{0} \varepsilon_{r} E{ }$$

9 $$J = \sigma E + j\omega D$$

10 $$\nabla \cdot J = 0$$

By combining these equations, Eq. (11) can be obtained:

11 $$\nabla \cdot \left[ {\left( {\sigma + j\omega \varepsilon_{0} \varepsilon_{r} { }} \right)\nabla V} \right] = 0$$

where *J*, σ, *E*, ω, *D* *and* *V* are electric current density, electrical conductivity, electrical filed, angular frequency, electric displacement field, and electrical voltage, respectively. The $\varepsilon_{0}$ is the relative permittivity of air and $\varepsilon_{r}$ is the relative permittivity of the fluid respected to the air. When alternative current (AC) voltage is applied, both real and imaginary terms are created in *J* which will be changed by the frequency. By solving these equations, the electric field ($E$) is calculated in the fluid. In this study, only the variable *E* is important as it has a direct influence on the amount of DEP force. Electrical potentials of 1 and -1 were used as a boundary condition for electrodes. All other boundaries were assumed to be insulated (glass and PDMS walls). The continuity condition was assumed for the electrodes and fluid interface. Triangular elements were chosen to mesh the geometry. The element size was finer near the electrodes’ boundaries, specifically the edges, and became coarser at regions further from the electrodes (see Supplementary Fig. S2 online).

### Fabrication of micro-separator

The proposed DEP device includes a glass substrate, which is patterned with gold electrodes, bonded to a PDMS channel. A layer of 25 nm chromium followed by an 80 nm layer of gold is sputtered using a magnetron sputtering system (Angstrom Sciences Inc.). To pattern the electrodes, a standard photolithography technique is used: first, a layer of photoresist S1805 (MicroChem Corp.) was spin-coated at 3,000 rpm for 30 s on the glass and baked at 115 °C for one minute. Next, the glass was exposed to ultraviolet (UV) light for 8 s, under the designed mask. The fabrication was then followed by washing away the exposed photoresist by rinsing the glass with the MF-319 developer (MicroChem Corp.). Next, gold and chromium etchants are used to pattern the electrodes. Finally, the remaining photoresist is stripped away in the photoresist remover 1165. To fabricate the PDMS channels, SU8 molds are fabricated in the cleanroom. Based on the desired height SU8 2025 or SU8 2075 is spin-coated on a silicon wafer. Following the guideline provided by MicroChem, the wafer is soft-baked at 65 and 95 °C. Afterward, it is exposed to the UV light and baked again. Subsequently, it is soaked in the SU8 developer and patterned. Finally, after washing with isopropanol and hard baking for 30 min at 150 °C, the mold is ready. A proper amount of PDMS and its curing agent are mixed with a proportion of 10 to 1 (w/w) and poured into the mold. After removing the bubbles in the desiccator and curing in the oven at 80 °C, the PDMS channel is peeled off, and the inlets and outlets are punched. Finally, the PDMS and glass are surface treated with oxygen plasma and bonded together (see Supplementary Fig. S3 online).

### Sample preparation and experimental procedure

The channel is washed with a solution of deionized (DI) water and 0.1% TWEEN 20 for about 30 min. The same solution is used as the sheath flow. The sample consists of the DI water, 0.1% (v/v) TWEEN 20, the 2% (v/v) polystyrene particles, and 20% (v/v) glycerol for density adjustment of the particles with the solution. The conductivity of the solution is measured as 20.3 μS/cm. The permittivity of the polystyrene particles and DI water is 2.4 and 76.4, respectively^53^. The permittivity of the solution is assumed to be the same as the DI water.

**Figure 5:**
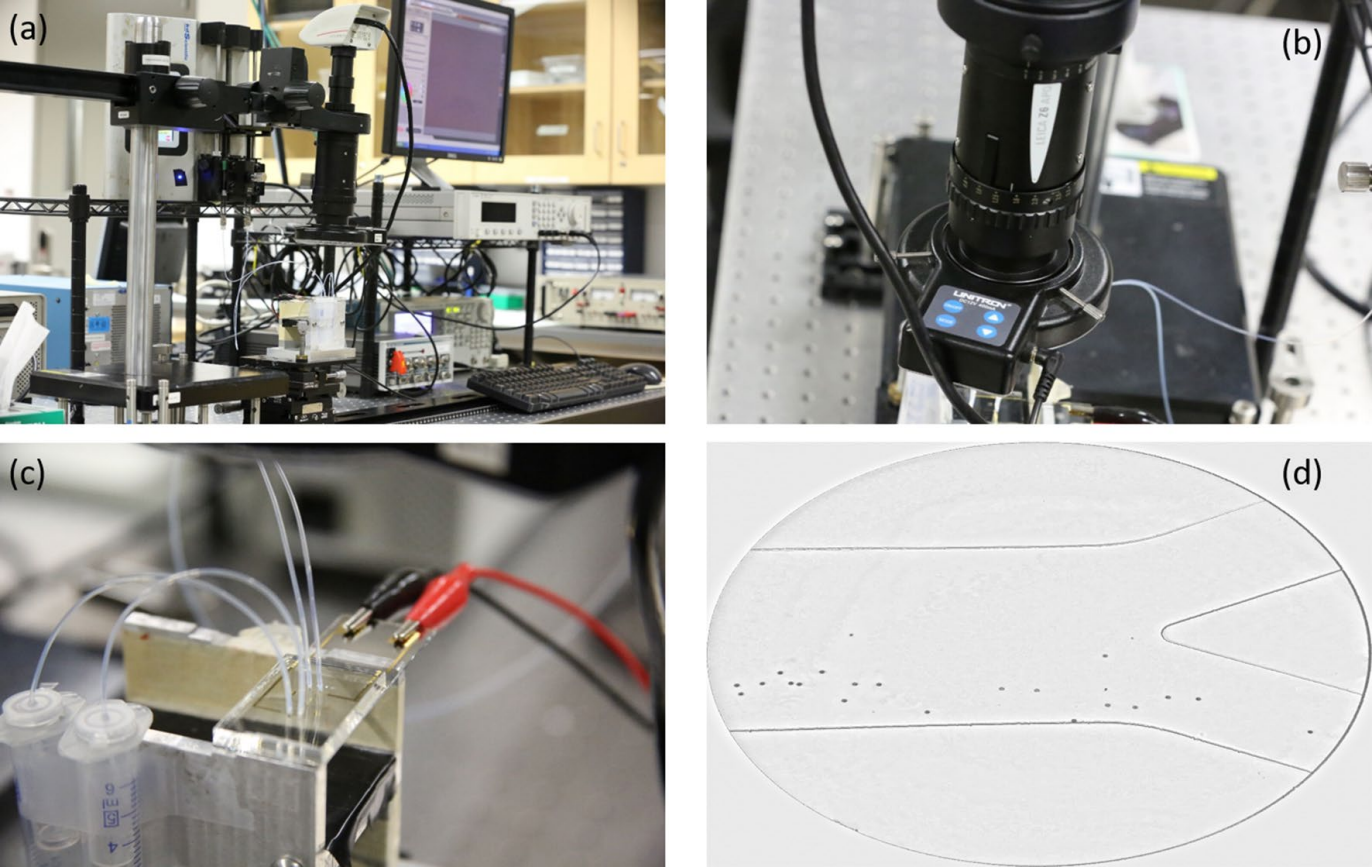
(**a**) The experimental setup including (**b**) an APO microscope, (**c**) a close-up view of the chip. The alligator clips and the inlet and outlet tubes are connected to the chip. The outlet solutions are collected for further analysis. (**d**) The polystyrene particles under the effect of the DEP force are guided effectively towards the target outlet.

A syringe pump (KD-scientific) is used to inject the particles and sheath flow through 1-mL syringes (Hamilton gastight) into the micro-channel. The proper voltage and frequency are applied to the electrodes by a signal function generator. Depending on the geometry of the channel and injected particles, the appropriate flow rate is applied through the syringe pump. A microscope (Leica) is used to observe the trajectory of the particles. The experimental setup and an example of the polystyrene particles, effected by the DEP, moving toward the Target Outlet are shown in Fig. 5a–d.

## Supplementary information

Supplementary file1 (PDF 476 kb)
