# Supplementary material for: Parametric study on the geometrical parameters of a lab-on-a-chip platform with tilted planar electrodes for continuous dielectrophoretic manipulation of microparticles
Source: Sci Rep. 2020 Jul 16;10:11718. doi: 10.1038/s41598-020-68699-4 (PMC7366698; doi:10.1038/s41598-020-68699-4)
Supplement: Supplementary file 1 — Supplementary file1 (PDF 476 kb) [file 41598_2020_68699_MOESM1_ESM.pdf]

Supplementary Information

Parametric study on the geometrical parameters of a lab-on-a-chip platform with tilted planar electrodes for continuous dielectrophoretic manipulation of microparticles

Arash Dalili[a], Erfan Taatizadeh[ab], Hamed Tahmooressi[a], Nishat Tasnim[a], Pamela Inés Rellstab-Sánchez[a], Matthew Shaunessy[a], Homayoun Najjaran[a], and Mina Hoorfar*[a]

[a] *School of Engineering, Faculty of Applied Science, The University of British Columbia, Kelowna, BC Canada V1V 1V7. E-mail: mina.hoorfar@ubc.ca*

[b] *School of Biomedical Engineering, Faculty of Applied Science | Faculty of Medicine, The University of British Columbia, Vancouver, BC Canada V6T 1Z3*

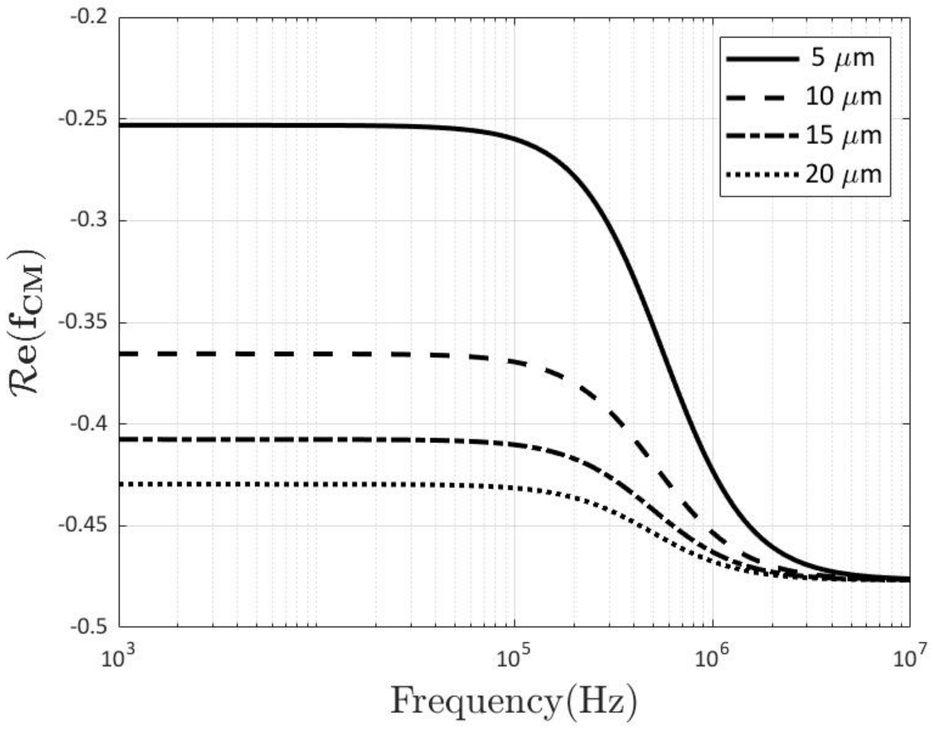

Figure S1.The CM factor for polystyrene particles as a function of the frequency of the applied electric field [drawn with MATLAB R2019 (https://www.mathworks.com)]

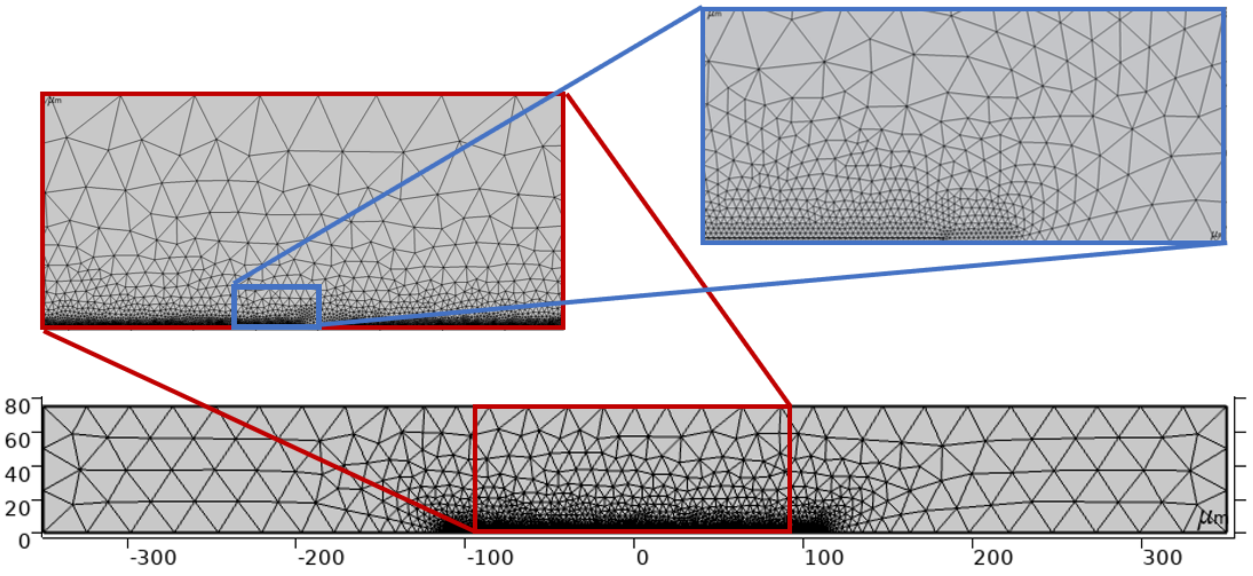

Figure S.2 An example of the geometries used for the simulation of the electric field. Traiangular elements are used to mesh the geometry using COMSOL Multiphysics.

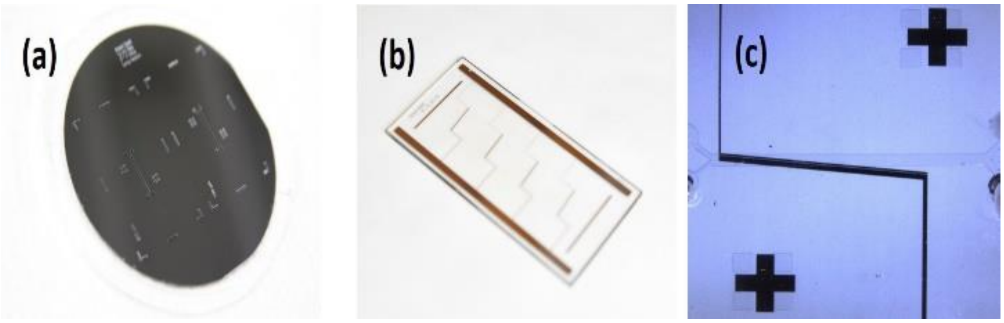

Figure S3. (a) The SU8 mold fabricated on a silicon wafer in the cleanroom. (b) A sample of the chips fabricated by photolithography. (c) The final chip under the microscope.
